# Prevalence and genetics of “de novo” MEN2 syndromes

**DOI:** 10.1210/clinem/dgaf171

**Published:** 2025-03-25

**Authors:** Roberta Casalini, Cristina Romei, Valeria Bottici, Virginia Cappagli, Valeria Tascini, Antonio Matrone, Alessandro Prete, Raffaele Ciampi, Teresa Ramone, Rossella Elisei

**Affiliations:** Department of Clinical and Experimental Medicine, Unit of Endocrinology, University Hospital of Pisa, 56100 Pisa, Italy; Department of Clinical and Experimental Medicine, Unit of Endocrinology, University Hospital of Pisa, 56100 Pisa, Italy; Department of Clinical and Experimental Medicine, Unit of Endocrinology, University Hospital of Pisa, 56100 Pisa, Italy; Department of Clinical and Experimental Medicine, Unit of Endocrinology, University Hospital of Pisa, 56100 Pisa, Italy; Department of Clinical and Experimental Medicine, Unit of Endocrinology, University Hospital of Pisa, 56100 Pisa, Italy; Department of Clinical and Experimental Medicine, Unit of Endocrinology, University Hospital of Pisa, 56100 Pisa, Italy; Department of Clinical and Experimental Medicine, Unit of Endocrinology, University Hospital of Pisa, 56100 Pisa, Italy; Department of Clinical and Experimental Medicine, Unit of Endocrinology, University Hospital of Pisa, 56100 Pisa, Italy; Department of Clinical and Experimental Medicine, Unit of Endocrinology, University Hospital of Pisa, 56100 Pisa, Italy; Department of Clinical and Experimental Medicine, Unit of Endocrinology, University Hospital of Pisa, 56100 Pisa, Italy

**Keywords:** MTC, *RET*, de novo mutations, MEN2

## Abstract

**Context:**

Hereditary medullary thyroid carcinoma (MTC) is an inherited syndrome accounting for 25% of MTC cases. It is caused by germline *RET* mutations, which can be inherited or occur de novo.

**Objective:**

This study aimed to define the prevalence and genetics of de novo MEN2 syndromes, which are not yet fully understood, and to characterize the parental origin of the *RET* de novo mutation.

**Methods:**

We selected 152 of 215 families with hereditary MTC. In de novo cases, we sequenced the wild-type and mutated alleles of the index cases and compared their single nucleotide polymorphism profiles with those of their parents. Digital droplet PCR was performed to determine the presence of mosaicism in both the index case and the parents.

**Results:**

In 24 of 152 (15.78%) families, the index case had a de novo mutation. Single nucleotide polymorphism analysis demonstrated that in all cases, the mutation occurred on the paternal allele. The absence of mosaicism supported the hypothesis that the mutation occurred during spermatogenesis. The mean age of fathers at the time of conception was, in some cases but not all, relatively advanced.

**Conclusion:**

The prevalence of de novo hereditary MEN2 syndromes was approximately 16%, including MEN2B, and around 9% for other phenotypes. All de novo cases were of paternal origin and likely resulted from an acquired alteration in sperm DNA. The possible role of advanced paternal age in promoting de novo mutations could not be ruled out.

Hereditary medullary thyroid carcinoma (MTC) is an autosomal dominant inherited syndrome that accounts for approximately 25% of all MTC cases (1-3). Based on clinical manifestations, 3 different syndromes can be recognized: Multiple Endocrine Neoplasia type 2A (MEN2A), type 2B (MEN2B), and Familial Medullary Thyroid Carcinoma (FMTC). MEN2A and MEN2B are rare genetic disorders characterized by the presence of MTC and other endocrine tumors. In particular, neoplasia of the parathyroid and adrenal glands occurs in 30% and 50% of MEN2A patients, respectively, with 10% of cases presenting with interscapular cutaneous lichen amyloidosis (4, 5). Also, ∼50% of MEN2B patients can develop adrenal gland neoplasia but not parathyroid neoplasia. They also have other distinctive features, such as marfanoid habitus, mucosal neuromas, ganglioneuromas, megacolon, corneal hypertrophy, and skeletal alterations (6). Although still controversial (7), the definition of FMTC implies that no other clinical endocrine manifestations are associated with MTC and that MTC alone is present in several other family members (8). MTC, which is always present in all 3 syndromes, is the only malignancy and can be very aggressive, particularly in those with MEN2B (6). Other tumors are most commonly benign although pheochromocytoma can be occasionally metastatic (9, 10).

Hereditary MTC is primarily caused by germline mutations in the *RET* (rearranged during transfection) proto-oncogene, which encodes a tyrosine kinase receptor that, when mutated, becomes constitutively activated, leading to C-cell proliferation and tumor transformation. These mutations account for ∼95% to 98% of all hereditary MTC cases, as reported by Wells et al (11).

Generally, in hereditary syndromes, mutations can be either inherited or de novo. Inherited mutations are passed down from 1 of the parents, whereas *de novo* mutations can occur either during gametogenesis or postzygotically (12). It is well known that most MEN2B cases are de novo (6, 13, 14), as are some MEN2A cases (15). However, the prevalence of de novo cases among inherited MEN2 syndromes is still unknown.

The present study aimed to define the prevalence of de novo MEN2 syndromes in a large series of hereditary MTC kindreds and to verify whether the *RET* de novo mutation always occurs on the paternal-derived allele, as reported in the few cases described so far (13, 16), or if there could be cases of maternal derivation.

## Materials and Methods

### Patients

A total of 215 families with hereditary MTC (ie, 137 with FMTC, 58 with MEN2A, 1 with MEN2A/2B (17), and 19 with MEN2B), followed at the endocrinology unit of the University Hospital of Pisa, Italy, were analyzed to establish the hereditary or de novo origin of the disease in the index cases. To determine the de novo or inherited status of the *RET* germline mutation, the blood of both parents of the index cases had to be available for genetic analysis. The epidemiological data of the index cases and their parents were also collected.

All screened subjects provided written informed consent for the *RET* genetic screening and to participate in the present study, which was approved by the institutional review board and the “Comitato Etico Regionale per la Sperimentazione Clinica della Regione Toscana (CEAVNO)” Prot 14387_ELISEI (20/04/2022).

## Methods

### Identification of the Parental Origin

To determine the parental origin of the *RET* germline-activating mutation, we selectively sequenced the wild-type and mutated alleles of the index case and compared the single nucleotide polymorphism (SNP) profiles of each allele with those obtained from the parents. Specifically, we investigated the SNP profiles in the amplified regions of introns 11-12 for cases with a *RET* germline mutation in exon 11, introns 15-16 for cases with the M918T mutation in exon 16, and introns 9-10 for cases with a *RET* germline mutation in exon 10. Genomic DNA was extracted from the blood of the index cases and parents using the automated system QIAcube Connect (QIAGEN, Hilden, Germany). Cases were considered “informative” or “noninformative” based on the identification of heterozygous or homozygous SNPs in the index case, respectively.

### SNP profile of introns 11-12 and 9-10

**Figure 1.**
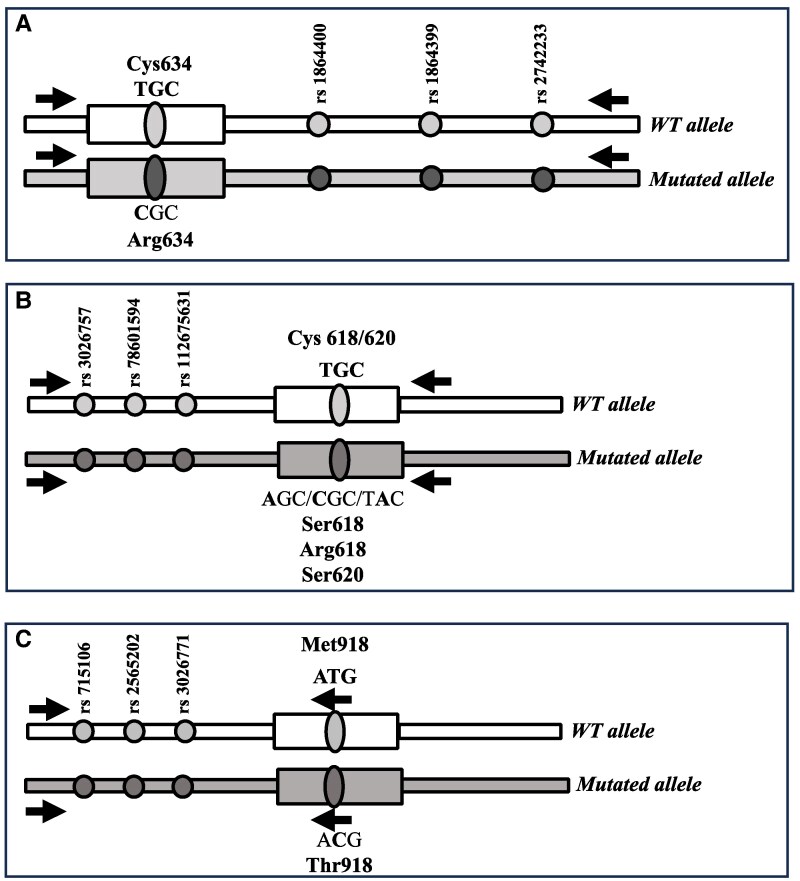
Schematic representation of the investigated SNPs and the primer positions. (A) SNPs analyzed in intron 11-12 to evaluate the de novo mutation at codon 634. (B) SNPs analyzed in intron 9-10 to evaluate the de novo mutation at codons 618 and/or 620. (C) SNPs analyzed in intron 15-16 to evaluate the de novo mutation at codon 918.

A region including exon 11 and part of intron 11-12 (1173 bp) of index cases with a *RET* germline mutation in exon 11, and a region including exon 10 and intron 9-10 (902 bp) of index cases with a *RET* germline mutation in exon 10 (Fig. 1, panels A and B), were amplified using the following primers: forward 5′-ACACCTCCATGGCCACTTC-3′ and reverse 5′-TAGGAAAGCTGGTGGGGAAG-3′ for intron 11-12, and forward 5′-AACTTCTCCACCTGCTCTCC-3′ and reverse 5′-CAATTTCCTCCCTTGTTGG-3′ for intron 9-10. The DNA of both the index cases and their corresponding parents was amplified. PCR products derived from the index case were submitted to gene cloning using the TA Cloning Kit (cat. K203001), according to the manufacturer's instructions (Life Technologies, Carlsbad, CA, United States). Index cases with homozygous SNPs were considered noninformative. Specifically, the PCR product was cloned into the linearized pCR 2.1 vector and used to transform One Shot Top 10 F′ cells. Antibiotic selection and white/blue colony screening were used to identify recombinant colonies, which were then grown in LB Broth medium. Plasmid DNA was prepared from cultured cells using the QIAprep Spin Miniprep Kit (Qiagen, Hilden, Germany) and sequenced using PCR primers.

### SNP profile of intron 15-16

To selectively sequence the wild-type and mutated alleles of index cases with the activating germline M918T mutation (Fig. 1C), degenerate primers were designed: a forward primer (5′-AAGCCATTTGTGTTGCCAGG-3′) mapped to intron 15-16, and 2 different reverse primers recognized the wild-type and mutated alleles, respectively (wild-type: 5′-AAAAGGGATTCAATTGCCATC-3′ and mutated: 5′-GGGATTCAATTGCCGTCCAT-3′). The PCR product was 982 bp. Parents were amplified using both the wild-type and mutated reverse primers; in the latter case, no amplification was obtained, indicating the specificity of the mutated reverse primer. The PCR protocol is available upon request.

### Identification of Parental Mosaicism

To analyze the presence of genetic mosaicism in both the index cases and the involved parent, digital droplet PCR (ddPCR) (BioRad, Hercules, CA, United States) was performed to determine the allelic frequency of the mutation, following the manufacturer's instructions, and using specific ddPCR assays for M918T (dHsaMDS309834335) and C634R (dHsaMDS2515168).

## Results

### Prevalence of de novo families in our series

**Figure 2.**
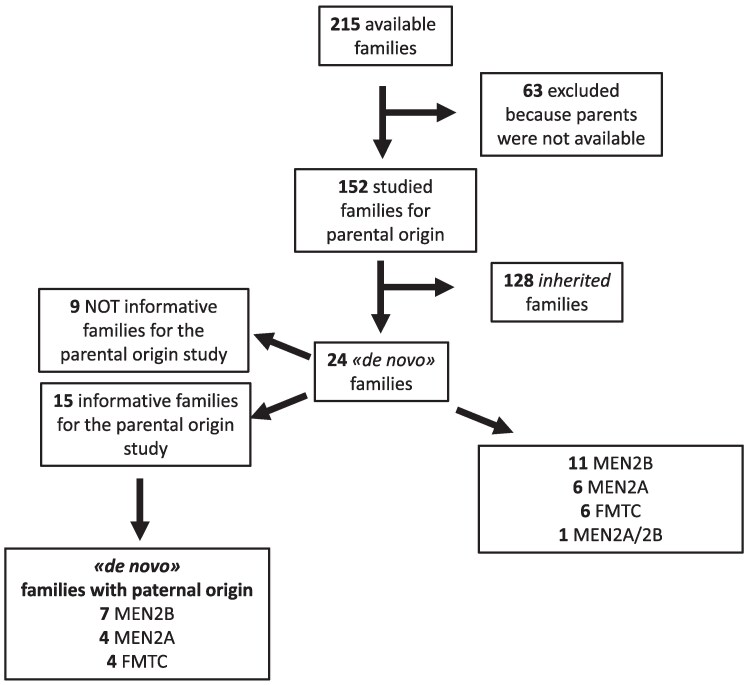
Selection of families included in the study and results of the SNP analysis.

Among the 215 families followed at the endocrinology unit of the University Hospital of Pisa from 1993 to 2024, blood samples from both parents were available for 152 cases, whereas 63 cases lacked this information. Although some of these 63 families had a long clinical history, and none of the parents of the affected subjects had ever developed the tumor, suggesting the possibility that they might be de novo, we decided to exclude them from the study to avoid potential bias (Fig. 2).

Twenty-four of 152 (15.78%) families were identified as de novo, as the *RET* germline mutation was present in the index cases but not in their parents. In contrast, 128 (85.33%) families were inherited, as the *RET* germline mutation in the index case was also present in 1 of the parents. As reported in Table 1, the key features of patients with de novo *RET* mutations were significantly different compared to index cases of inherited syndromes, particularly in terms of age at diagnosis, phenotype, and mutation distribution. A slight, but not significant, difference was observed when comparing the age of fathers at conception. No differences were observed based on sex. Specifically, among the 24 de novo families, 6 (25%) were FMTC, 6 (25%) were MEN2A, 11 (45.8%) were MEN2B, and 1 (4.17%) was a peculiar MEN2A/MEN2B case previously described (15). When we considered the prevalence of de novo cases across the 3 different phenotypes, we found that 11/11 (100%) MEN2B, 14.6% (6/41) of MEN2A, and 6.0% (6/99) of FMTC were de novo cases. After excluding the MEN2B cases, which are known to be almost always de novo, the prevalence of de novo mutations in both MEN2A and FMTC was 8.6%. Among the 128 inherited families, 93 (72.6%) were FMTC and 35 (27.3%) were MEN2A. No MEN2B or MEN2A/MEN2B families were found in the inherited group.

**Table 1.** Comparison between the salient features of patients with de novo mutations compared with patients with inherited syndromes

	De novo (n = 24)	Inherited (n = 128)	*P*
**Age at diagnosis (y)**			
All	22.17 ± 11.07	43.91 ± 15.65	<.0001
No MEN2B	27 ± 10	43.91 ± 15.65	.0046
**Sex**			
Female	15	80	
Male	9	48	NS
**Phenotype**			
MEN2A	6	35	
MEN2A/2B	1	0	< .0001
MEN2B	11	0	
FMTC	6	93	
***RET* germline mutations**			
M918T	11	0	
C634Any	8	31	
Del ex 11	1	0	< .0001
Cys ex 10	4	25	
Other	0	72	
Age of father at conception	34.9 ± 5.1	32.1 ± 6.8	NS

Abbreviations: FMTC, Familial Medullary Thyroid Carcinoma; MEN, Multiple Endocrine Neoplasia; NS, not significant

The *RET* germline de novo mutations are reported in Table 2. Except for the typical M918T mutation in MEN2B and the peculiar mutation in the MEN2B/MEN2A case, all the other mutations were cysteine alterations. No de novo cases due to noncysteine mutations were found.

**Table 2.** Germline RET mutations in the “de novo” MEN2 families

*RET* “de novo” mutation	“de novo” families		Total
	Paternal origin (n)	Noninformative (n)	
M918T	7	4	11
C634R	3	3	6
Glu632_Leu633del	0	1	1
C634Y	1	0	1
C634G	1	0	1
C620S	1	1	2
C618S	1	0	1
C618R	1	0	1
Total	15	9	24

### SNPs analysis in index cases and their corresponding parents

Sequence analysis of intron 11-12, intron 15-16, and intron 9-10 was performed to determine whether the *RET* mutation occurred on the paternal or maternal allele in families with the M918T mutation in exon 16, cysteine mutations in exon 11, and exon 10.

**Figure 3.**
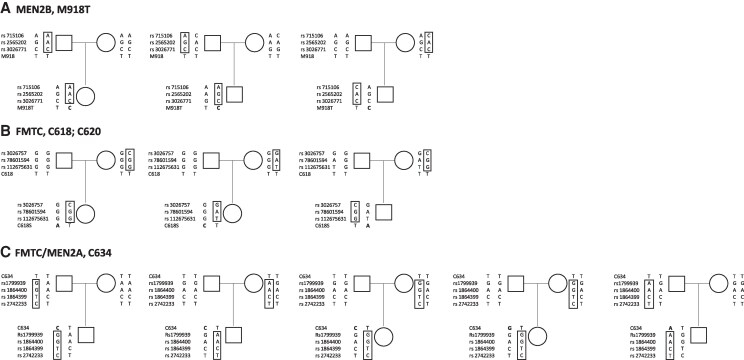
Pedigree of the de novo informative families, indicating that the index case's mutated allele derives from the father. The mutated nucleotide is shown in bold in each pedigree; the SNP’s profile in the box indicates the informative allele. (A) MEN2B families with M918T *RET* mutation; (B) FMTC families with *RET* mutation at codon 618 and 620; (C) FMTC/MEN2A families with *RET* mutation at codon 634.

Among the 11 de novo families with the M918T germline mutation, the analysis of SNP distribution in the parents and index cases demonstrated that the mutation occurred on the paternal allele in 7 of 7 informative cases (Fig. 3A). The remaining 4 families were found to be noninformative.

The 2 families with mutations at codon 618 (C618S and C618R), as well as the family with the C620S mutation, were also found to have a paternal origin, whereas a second family with the C620S RET mutation was noninformative (Fig. 3B). Among the 9 families with a cysteine mutation in exon 11 (C634R n = 6; C634Y n = 1; p.Glu632_Leu633del n = 1; C634G n = 1), the analysis of SNP distribution in the parents and in the index case demonstrated that the mutation occurred on the paternal allele in 5/5 informative cases (Fig. 3C), whereas 4 cases were noninformative for the identification of the parental origin. A summary of the parental origin of the 24 de novo families is shown in Fig. 2.

### Evaluation of the age at the time of conception of the involved parent

The overall mean age at conception of fathers of index cases with a de novo mutation was 34.9 years ± 5.17 years (range: 23-42 years). However, according to the Italian National Statistical data (Istituto Italiano di Statistica: https://www.istat.it/), there has been a significant increase in paternal age at the time of the first child, rising from 25 years in the 1990s to the current 35.8 years. We distinguished the paternal age of the de novo cases born before and after 2000. The analysis showed that the paternal age before and after 2000 was 34.46 ± 5.6 and 36.2 ± 3.2, respectively.

### Analysis of genetic mosaicism in the involved parent

ddPCR analysis of the de novo mutation in the index cases revealed a mean allelic frequency of 47.3% (range: 37-56), as expected in the case of genetic homogeneity. Conversely, the fathers analyzed for potential mosaicism were all completely negative for the germline *RET* mutation, even with a very sensitive method like ddPCR.

## Discussion

De novo mutations represent a very important source of genetic variation, and when they involve disease-causing genes, they induce the development of a genetic syndrome (18). Among others, dominant de novo mutations are often the cause of rare genetic diseases, such as Down syndrome, which is due to a de novo trisomy of chromosome 21 (19, 20), Apert syndrome, and Noonan syndrome, whose causative genes belong to the RAS/MAPK signaling pathway (21, 22).

The majority of MEN2B and some cases of MEN2A have been described as de novo syndromes caused by *RET* de novo mutations (6, 13, 15, 16, 23, 24), but so far, no data on the prevalence of de novo *RET*-mutated syndromes have been reported, with the exception of a study describing about 84% of de novo MEN2B in a large multicentric study dedicated to MEN2B (25). In our study, which was conducted on 1 of the largest series of hereditary MTC syndromes followed at a single tertiary referral center, we established that almost 16% of these syndromes are de novo. In particular, 100% of MEN2B and about 9% of the MEN2A/FMTC phenotypes were found to be de novo. To our knowledge, this prevalence has never been deeply studied before, as the only study reporting the prevalence of de novo cases in the MEN2A/FMTC phenotype has the bias of including cases where the parents were not investigated, whereas we specifically selected only kindreds for which this information was available (15). The high prevalence of de novo syndromes in MEN2B, compared to other hereditary forms of MTC, could be explained by the severity of MEN2B (6, 25, 26), which often impairs the quality of life of affected individuals, who rarely reach fertility age in good health. Only in recent years, thanks to new targeted therapies (27), have MEN2B-affected patients been able to survive with a good quality of life and retain the ability to reproduce. In fact, the diagnosis of MEN2B is often made too late, when MTC is already advanced, as the other clinical manifestations typical of this syndrome, although clinically evident, are not recognized early enough (26). In contrast, patients affected by MEN2A or FMTC usually present with a less severe phenotype (7), and most of these patients reproduce, leading to several offspring. We could hypothesize that this accounts for the lower prevalence of de novo MEN2A/FMTC cases. Another possible explanation is that codon 918 is likely the most susceptible site in the *RET* gene to mutation, as demonstrated by the greater prevalence of the M918T mutation in sporadic forms (28). Moreover, both somatic MTC mutations and de novo MEN2B mutations are acquired mutations, likely facilitated by fragility at that codon in the DNA.

It has been reported that patients with MEN2B resulting from a germline *RET* mutation, whether de novo or inherited, are significantly younger at thyroidectomy than sporadic patients with a somatic M918T mutation (29). In our series, the mean age at diagnosis for de novo cases with a germline mutation was also significantly lower than for sporadic patients harboring the same somatic mutation. This phenomenon was observed not only in those with M918T (17.11 ± 6.99 years vs 49.07 ± 13.74 years) but also in those with a cysteine mutation (25.5 ± 9.58 years vs 57.11 ± 14.21 years), reinforcing the concept that the presence of a *RET* germline mutation contributes to more rapid tumor development, with clinical evidence appearing at a younger age.

In our series, all *de novo* mutations were found to be of paternal origin. With the exception of a single report describing a M918T de novo mutation on the maternal allele (30), literature data support the evidence that de novo mutations are almost exclusively of paternal origin (13). The occurrence of de novo mutations on the paternal allele is also a frequent mechanism in many de novo syndromes (31), such as Costello (32), Noonan (21), and Apert syndrome (22). The most significant explanation for this phenomenon is that spermatogonial cells continue to divide throughout a man's life. The continued divisions of spermatogonia allow the progressive accumulation of DNA errors that ultimately could result in sperm cells with unexpected gene mutations (33). In contrast, oocytes do not divide during a woman's life, and their number decreases with age. It is therefore unlikely that mutations occur randomly in these cells. Nevertheless, it cannot be completely excluded that the de novo mutation could occur during the early stages of zygote development. Indeed, it has been reported that de novo mutations may arise as postzygotic events in the proband (34). These authors found that 6.5% of presumed germline de novo mutations were, in fact, present at a low allelic frequency in the blood of the offspring and were therefore likely to have occurred postzygotically (34). In our series, the allelic frequency of all de novo mutations in the index cases was approximately 50%, as confirmed by ddPCR, suggesting that, in our cases, the de novo mutations most likely did not occur postzygotically.

The de novo mutations have also been shown to be caused by low-level mosaicism in 1 of the parents (34, 35). In the present study, we did not find any mosaicism in the father's blood, suggesting that this phenomenon is unlikely to be causative for the onset of our de novo cases. This hypothesis is further supported by the evidence that, among our de novo families, as well as in others reported in the literature (15, 23), no *RET*-mutated subjects were identified among the siblings of the index case, which would be expected in the case of parental mosaicism.

It is also known that older paternal age is a risk factor for the occurrence of de novo mutations, as the likelihood of new mutations increases with age (36), primarily from errors made by DNA repair mechanisms (37, 38). In our series, the overall mean age at conception of fathers of index cases with de novo mutations was 34.9 years, which is similar to the age of males currently having their first child, but older than that of males who had their first child in the 1990s (data from Istituto Italiano di Statistica: https://www.istat.it/). This finding was confirmed when we distinguished paternal age according to the birth year of the index cases (ie, before and after 2000). Specifically, for cases born before 2000, fathers were 10 years older than expected, whereas for cases born after 2000, fathers' ages were similar to those of the general contemporary population. Based on these results, it is difficult to correlate paternal age with the occurrence of the de novo mutation, at least in this series.

In conclusion, this study, for the first time, reported the prevalence of de novo hereditary cases among the MEN2 syndromes, which was approximately 16% when including MEN2B and about 9% when restricted to the MEN2A/FMTC phenotypes. This highlights the necessity of screening for genetic *RET* alterations in all apparently sporadic MTC cases. Moreover, we confirmed that all de novo cases were of paternal origin and likely from an acquired alteration in spermatogonial DNA. The advanced age of the father remains a possible, but controversial, influencing factor in facilitating the occurrence of de novo mutations.

## Data Availability

Original data generated and analyzed during this study are included in this published article or in the data repositories listed in References.

## Abbreviations

ddPCR: digital droplet PCR
FMTC: Familial Medullary Thyroid Carcinoma
MEN: Multiple Endocrine Neoplasia
MTC: medullary thyroid carcinoma
SNP: single nucleotide polymorphism
